# Disseminated iatrogenic upper gastrointestinal Kaposi sarcoma following prolonged steroid use in a patient with Crohn’s disease: a case report

**DOI:** 10.1186/s13256-026-05937-9

**Published:** 2026-03-12

**Authors:** Glory Makupa, Furaha Serventi, Leila Mwakipunda, Godwin Nnko, Edwin Liwa, Salum J. Lidenge, Ayesiga Herman, Lilian Gasper, Amos Mwasamwaja, Beatrice Kombole, Alex Mremi

**Affiliations:** 1 Cancer Care Centre, Kilimanjaro Christian Medical Centre, Moshi, Tanzania; 2 School of Medicine, KCMC University, Moshi, Tanzania; 3 Kilimanjaro Clinical Research Institute, Moshi, Tanzania; 4 Department of Pathology, Arusha Lutheran Medical Centre, Arusha, Tanzania; 5 Department of Pathology, Kilimanjaro Christian Medical Centre, Moshi, Tanzania; 6 Clinical Research Training and Consultancy Unit, Ocean Road Cancer Institute, Dar es Salaam, Tanzania; 7 Clinical Oncology Department, Muhimbili University of Health and Allied Sciences, Dar es Salaam, Tanzania; 8 Aga Khan University Hospital, Nairobi, Kenya; 9 Muhimbili National Hospital, Dar es Salaam, Tanzania

**Keywords:** Iatrogenic Kaposi sarcoma, Human herpesvirus-8, Prolonged steroid use, Crohn’s disease, Case report

## Abstract

**Background:**

Kaposi sarcoma is a malignant vascular neoplasm caused by human herpesvirus-8. It is mostly seen in immunocompromised individuals, particularly those with human immunodeficiency virus/acquired immunodeficiency syndrome or organ transplants. Cutaneous and mucosal Kaposi sarcoma manifestation is common; however, Kaposi sarcoma may involve the visceral organs. Iatrogenic or drug-induced Kaposi sarcoma may develop in immunocompromised patients undergoing immunosuppressive medication. It is quite uncommon for patients with inflammatory bowel diseases on immunosuppressive medication to develop intestinal Kaposi sarcoma. This case report describes the clinical presentation of steroid-induced disseminated upper gastrointestinal Kaposi sarcoma in a patient with underlying Crohn’s disease and examines the management approach, associated challenges, and clinical outcome.

**Case presentation:**

We report a rare case of iatrogenic Kaposi sarcoma in a 39-year-old African male patient with Crohn’s disease undergoing long-term corticosteroid treatment. Patient history, clinical examination, and investigations revealed mucosal Kaposi sarcoma with human herpes virus-8 positivity. This prompted discontinuation of corticosteroids with subsequent transition to infliximab a biologic agent. During the course of treatment with infliximab, the patient presented with extensive Kaposi sarcoma and was started on systemic chemotherapy with improvement.

**Conclusion:**

This case underscores the importance of maintaining a high index of suspicion for Kaposi sarcoma in patients on chronic immunosuppressive therapy who present with unusual skin or mucosal lesions.

## Introduction

Kaposi sarcoma (KS) is an angioproliferative malignancy associated with human herpes virus 8 (HHV-8) [1]. Primary infection with HHV-8 can either be asymptomatic, minimally symptomatic, or symptomatic [2]. KS is one of the manifestations of symptomatic HHV-8 infection and occurs in four epidemiologic forms: classic KS and indolent form, which occurs in Eastern European, Mediterranean, or Middle Eastern regions; endemic KS, which is an aggressive form seen in Equatorial Africa; iatrogenic and immunosuppression-related KS, occurring predominantly owing to exogenous immunosuppression mostly seen in organ transplant patients; and acquired immunodeficiency syndrome (AIDS)-associated KS [3]. Prolonged use of steroids has been reported to be one of the causes of iatrogenic KS [4–8]. This may manifest clinically as cutaneous, mucosal, or visceral lesions [3]. Cutaneous lesions may present as patches, plaques, or nodules, and the purple appearance of these lesions is attributed to their highly vascular nature [3]. The most common iatrogenic noncutaneous sites are the oral cavity, gastrointestinal tract, and respiratory system [9, 10]. Lesions involving the gastrointestinal tract may be asymptomatic or may present with nausea and vomiting, abdominal pain, iron-deficiency anemia, malabsorption, weight loss, upper or lower gastrointestinal bleeding, intestinal obstruction, and/or diarrhea [10]. Iatrogenic KS due to long-term use of immunosuppressive agents has been well documented in solid organ transplant recipients and patients with rheumatological, pulmonary, and hematological diseases undergoing corticosteroid therapy [4, 5, 9, 11]. However, reports of its occurrence in gastrointestinal diseases following prolonged immunosuppressive use are still limited.

Crohn’s disease is a chronic inflammatory condition that involves any segment of the gastrointestinal tract [12]. Symptoms manifest variably depending on the segment involved, but the main ones include abdominal pain, diarrhea (with or without gross bleeding), fatigue, and weight loss [12]. Treatment options include the use of medical therapy, such as steroids, aminosalicylates, immunomodulators, and biologic therapy, and/or surgery on the basis of the severity and anatomic location of disease [13].

We herein report a case of immunosuppression-related KS in a patient who had long-term use of steroids for the management of Crohn’s disease. It is plausible that the clinical manifestation of gastrointestinal (GI) KS symptoms was obscured by symptoms associated with Crohn’s disease, complicating timely recognition and diagnosis. This case highlights the importance of vigilance for the diagnosis of KS in patients on long-term immunosuppressive therapy and the effective use of chemotherapy in treating KS.

## Case presentation

A 39-year-old African male presented at our facility in 2024 with a history of Crohn’s disease diagnosed 8 years prior to attendance at our facility in 2024. Initial diagnostic colonoscopy at that time revealed stenosed anal canal and severely inflamed edematous rectal mucosa with deep ulceration and distorted architecture. Imaging and histopathology results demonstrated features of Crohn’s disease. He was started on sulfasalazine, an aminosalicylate, with no improvement. A biologic agent infliximab was later recommended but not initiated owing to cost constraints and unavailability.

He was then started on oral prednisolone in 2018 at a dose ranging from 0.4 mg/kg to 1 mg/kg, adjusted on the basis of the severity of flare symptoms. The patient had a history of herpes zoster in the fifth year of prednisolone use and was treated successfully with antiviral therapies.

**Fig. 1:**
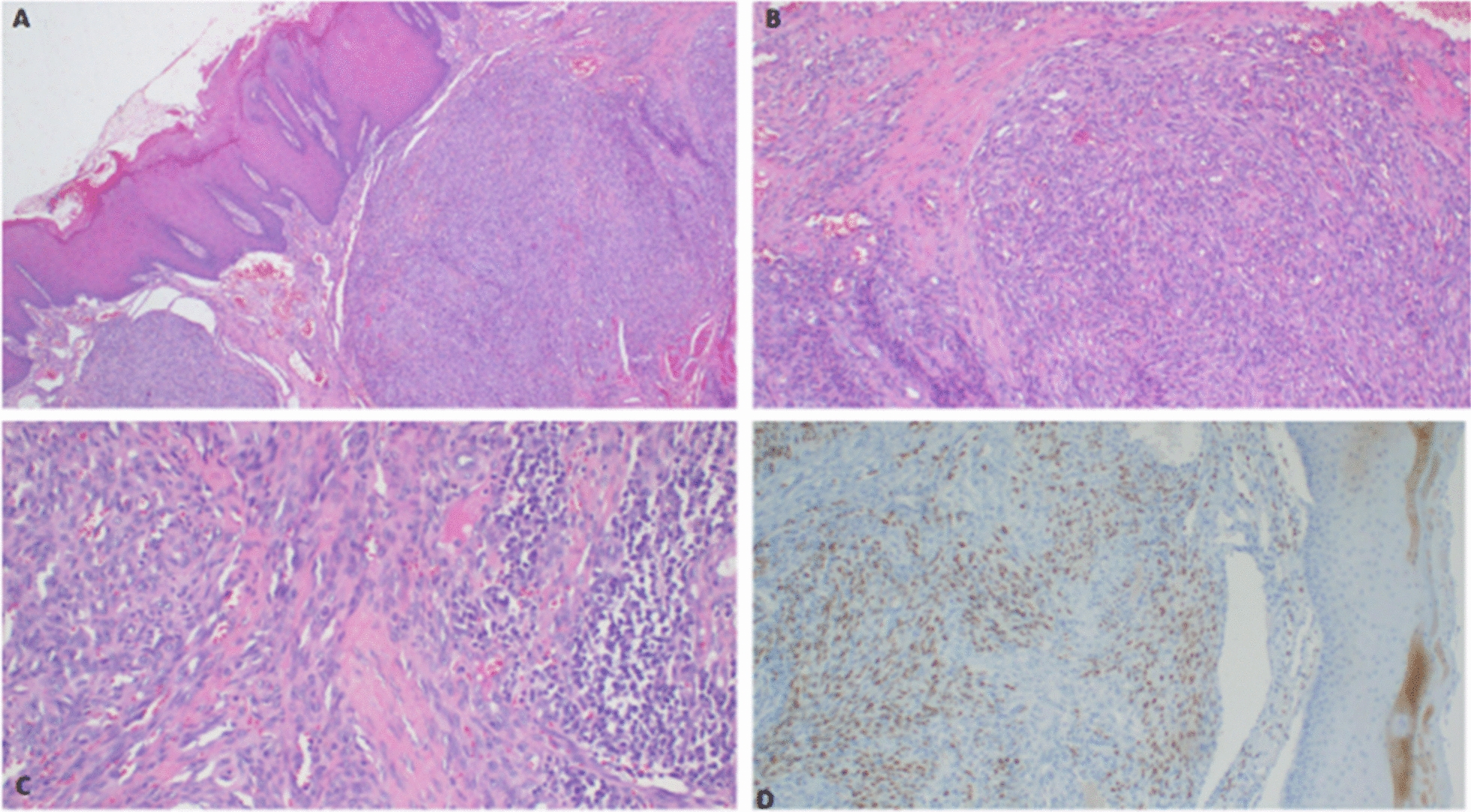
Histopathology of the tongue biopsy demonstrates sub-mucosal tumor nodules: hematoxylin and eosin (H&E) staining at 4× original magnification (**A**). The tumor is composed of slit like vascular spaces formed by spindled endothelial cells with minimal cytologic atypia and extravasation of red blood cells: H&E staining at 10× original magnification (**B**). The presence of plasma cells are evident: H&E staining at 20× original magnification (**C**). The tumor cells express immunostaining with anti-HHV-8 antibody: immunohistochemistry (IHC) at 10× original magnification (**D**)

During his sixth year of prednisolone use, he noticed a painless, hyper-pigmented, violaceous tongue papule that was gradually increasing in size and associated with bleeding on touch. An excisional biopsy performed at a local hospital revealed histopathological findings consistent with KS characterized by a nodular tumor made up of atypical spindle-shaped cells with red blood cell (RBC) extravasation and positivity with HHV-8 (Fig. 1A–D). He was subsequently referred to our tertiary care center, equipped with cancer treatment services, for further evaluation and management.

**Fig. 2:**
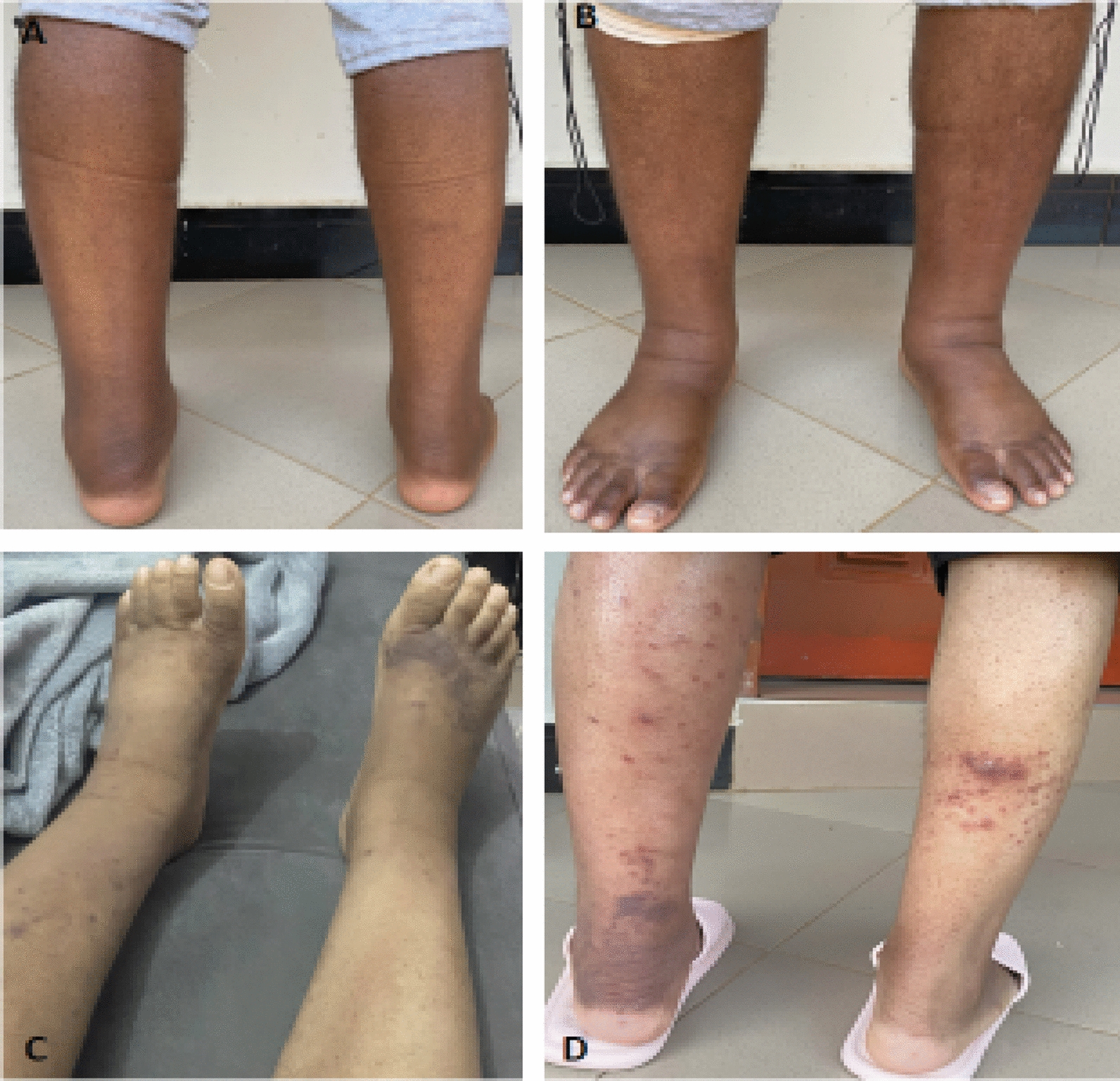
The bilateral lower limb edema (**A**, **B**) during use of steroids; multiple new hyperpigmented lesions on bilateral lower limbs associated with persistent wooden hard edema (**C**, **D**)

At our center, the patient’s medical history revealed frequent episodes of passing loose stool accompanied by the presence of gross blood associated with abdominal pain, reduced oral intake, and weight loss. These symptoms were temporarily relieved by prednisolone. On physical examination, he had a puffy (Cushing-like) face with bilateral lower limb edema grade 3 (Fig. 2A, B). On oral examination, there was post-excisional biopsy status of the papule. Initial laboratory investigations revealed features of iron-deficiency anemia, low ferritin, normal trans-ferritin, and elevated C-reactive protein. Serology for human immunodeficiency virus and *Helicobacter pylori* stool antigen tests were negative. Imaging with chest X-ray and abdominal pelvic ultrasound were normal.

Virtual multidisciplinary tumor board discussion recommended the need to mitigate the immunosuppressive effects of prolonged corticosteroid use as a management of the oral KS. A monoclonal antibody infliximab was initiated on the basis of its superior efficacy and prolonged therapeutic response. Infliximab was prescribed at a dose of 250 mg intravenously daily on day 0, day 2, week 6, and every 8 weeks thereafter. The treatment course also included tapering prednisolone, iron supplementation, and nutritional optimization.

**Fig. 3:**
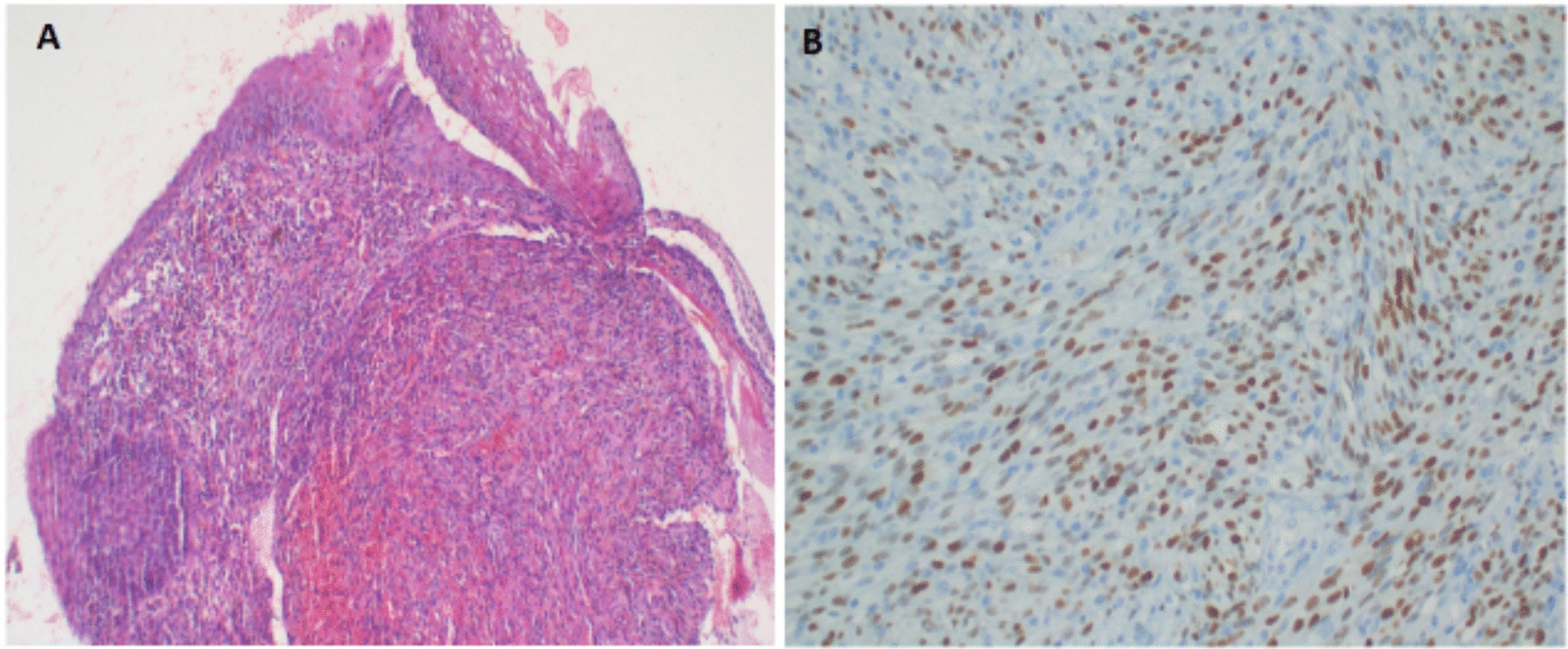
Esophageal biopsy section shows a nodular tumor composed of prominent spindle-shaped cells and slit-like spaces containing extravasated red blood cells: H&E staining at 10× original magnification (**A**). Immunohistochemistry highlighting a positive staining by HHV-8 (**B**)

After two initial doses of infliximab, the patient developed multiple new hyperpigmented lesions on bilateral lower limbs associated with persistent wooden hard edema (Fig. 2C, D). He also had epigastric pain, which was more exacerbated after taking a meal and during the night, associated with frequent loose stool. Esophagogastroduodenoscopy revealed multiple duodenal and esophageal lesions, which upon biopsy revealed KS with HHV-8 positivity (Fig. 3A, B). Contrast-enhanced chest, abdominal, and pelvic computed tomography (CT) scans had no radiologically visible pulmonary or other abdominal visceral involvement. Colonoscopy could not be performed at the facility owing to significant stenosis of the lower gastrointestinal tract, necessitating the use of a smaller caliber endoscope, which was unavailable.

**Fig. 4:**
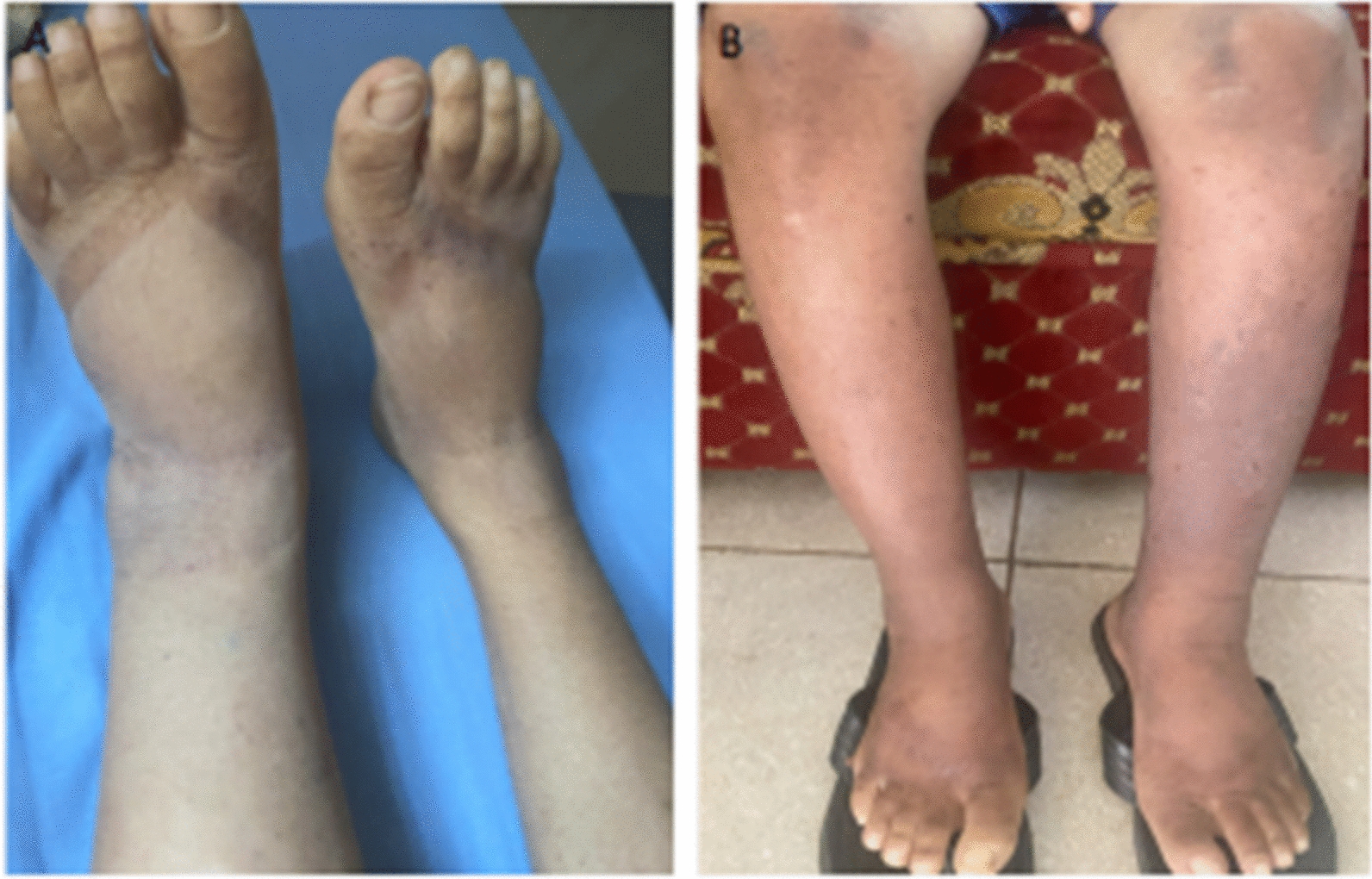
Resolution of bilateral lower limb lesions after chemotherapy and immunotherapy use

A diagnosis of extensive upper GI KS disease was instituted, and the patient was started on chemotherapy given the extensive disease. He received six cycles of paclitaxel at a dose of 135 mg/m^2^ intravenously every 3 weeks with significant improvement. The patient was monitored for chemotherapy-related toxicity through clinical assessments and routine complete blood counts and liver and renal function tests. The patient tolerated the chemotherapy well with only mild transient fatigue and no grade 3/4 hematologic or non-hematologic toxicities. By the completion of six cycles, lesions had nearly all resolved, with no new lesions or systemic complications (Fig. 4). To date, 6 months after completion of paclitaxel treatment, he remains free from signs and symptoms of KS. He reports improvement of bowel movement, with less frequent loose stool, not blood stained. He also has reduced abdominal pain and improved appetite. Table 1 presents the timeline and sequence of events in the case.

**Table 1:** Timeline of events in the patient’s case

Time point	Clinical presentation	Investigations and diagnostic findings	Management
2016	Gastrointestinal symptoms	Imaging, colonoscopy, and biopsy confirmed Crohn’s disease	Sulfasalazine initiated
2018	No improvement on sulfasalazine	Persistent disease clinically	Infliximab planned but unavailable Prednisolone initiated
2023	Presented with *Herpes zoster*	Clinical diagnosis	Treated with antivirals
April 2024	Hyperpigmented, violaceous tongue papule	Biopsy confirmed Kaposi sarcoma	Referred to cancer center
May 2024	Increasing GI symptoms, weight loss, and bilateral lower limb edema	HIV serology: negative Chest X-ray and abdominal pelvic ultrasound were normal	Multidisciplinary team advised tapering prednisolone, starting on infliximab owing to limited KS disease and optimize nutrition
July 2024	Presented with epigastric pain, multiple new hyperpigmented lesions on bilateral lower limbs	Esophagogastroduodenoscopy and biopsy: confirmed extensive upper gastrointestinal Kaposi sarcoma Chest, abdominal, and pelvic CT scan showed no radiologically visible visceral involvement	Started paclitaxel 135 mg/m^2^ intravenously every 3 weeks × six cycles
January 2025	Marked clinical improvement	No visible new lesions	Continue with infliximab and follow-up

## Discussion

This case highlights an uncommon but serious adverse event of long-term steroid therapy. KS in the context of steroid use in the treatment of Crohn’s disease is rare, emphasizing the need for vigilance when using steroids for Crohn’s disease. The overlap in clinical symptoms between Crohn’s disease and gastrointestinal KS may contribute to delayed recognition of KS.

The pathogenesis of iatrogenic KS secondary to long-term steroid use results from significant immunosuppression due to impaired production of pro-inflammatory cytokines, inhibition of free radicals and metalloproteases, and elevation of immunomodulatory prostaglandins [14]. This immunosuppressive state subsequently facilitates reactivation of human herpes virus 8 (HHV-8) infection [14, 15]. This viral reactivation was evidenced by the HHV-8 viral detection in biopsied tissues, coinciding with the appearance of characteristic violaceous mucosal and cutaneous lesions.

The use of several steroid agents in the treatment of autoimmune conditions has been implicated in the development of KS. Previous reports have also documented cases of KS associated with other steroids, including betamethasone, methylprednisolone, and triamcinolone [8, 16, 17]. Our patient was receiving prednisolone, an oral steroid, for an extended period. Prednisolone has also been used in the management of other autoimmune conditions, including rheumatological, pulmonary, and hematological diseases undergoing corticosteroid therapy [4, 5, 7, 9]. However, while KS has been reported in cases involving other organ systems, reports of prednisolone-associated KS in Crohn’s disease remain limited. Given the overlapping symptoms between Crohn’s disease and visceral KS, this case is particularly important as delayed recognition of KS in this patient population may lead to prolonged undiagnosed disease and subsequent complications.

In this present case, the patient had been on prednisolone for approximately 6 years, with doses ranging from 20 mg/day to 40 mg/day. Previous reports have documented varying durations of prednisolone use before the onset of KS. For instance, Endo *et al*. reported KS development after 1 year, Chen *et al*. at 7 months, Joo and Vincent *et al*. at 4 months, Yoo *et al*. at 10 weeks, Trattner *et al*. ranging from 3 to 36 months, and Manion *et al*. as early as 4 days [7, 8, 17–21]. However, our patient had been on steroids for an extended period, suggesting that gastrointestinal KS may have been present for a prolonged duration but remained undiagnosed owing to low index of suspicion for visceral KS because of the shared clinical features such as abdominal discomfort, nausea and vomiting, diarrhea, blood in stool, and anemia.

Similarly, reported steroid dosing in prior cases has varied significantly. Manion *et al*. described a dose of 60 mg per day, while Trattner *et al*. reported a wide range from 4 to 125 mg daily [17, 21]. Our patient self-adjusted his prednisolone dose from 20 to 40 mg per day over approximately 6 years on the basis of his flare symptoms. While there is no established correlation between steroid dose, duration, and the development of KS, this case underscores the importance of maintaining a high index of suspicion in patients receiving long-term steroids, particularly when treating conditions that share clinical features with KS and particularly in this population where the prevalence of HHV-8 is high [22]. We suggest early endoscopy and comprehensive imaging for patients with Crohn’s disease receiving steroids and even in limited cutaneous or mucosal disease to facilitate timely intervention.

Given the diagnosis of KS and its association with prolonged steroid use, we initially opted to discontinue prednisolone on the basis of current recommendations for management of steroid-induced KS and initiated infliximab, a monoclonal antibody known to have a more prolonged therapeutic response [13]. While previous reports have also documented cases of KS in patients receiving infliximab, we considered its potential benefit in this case [23, 24]. Infliximab was selected as an immunomodulatory agent owing to its superior efficacy compared with steroids and long-term remission in Crohn’s disease even after withdrawal [25, 26]. The decision was made within a multidisciplinary team, balancing the need to control Crohn’s disease against the potential risk of KS reactivation. Following cessation of steroids, transition to infliximab alongside chemotherapy, and with clinical monitoring, the patient demonstrated significant clinical improvement. Malnutrition is a common complication of Crohn’s disease resulting from impaired nutrient absorption, associated GI symptoms, and gut microbiota disturbances. Although malnutrition can contribute to impaired immune function that could result in KS, in this case, it was secondary to the underlying Crohn’s disease rather than a primary cause of immunosuppression.This case highlights the complexity of managing steroid-associated complications and underscores the need for individualized treatment strategies that balance disease control with the risk of adverse effects.

## Conclusion

The overlapping clinical features of Crohn’s disease and gastrointestinal KS may contribute to delayed KS recognition. Thorough history, examination, and targeted investigations are warranted especially in patients with long-term steroid use to reduce treatment-related complications. Generally, a high index of suspicion for KS in patients with long-term steroid use is important.

## Data Availability

The data that support the findings of this study are readily available.

## Abbreviations

AIDS: Acquired immune deficiency syndrome
GI: Gastrointestinal
H&E: Hematoxylin and eosin
HHV-8: Human herpesvirus-8
IHC: Immunohistochemistry
KS: Kaposi sarcoma
